# Associations between physical activity, long COVID symptom intensity, and perceived health among individuals with long COVID

**DOI:** 10.3389/fpsyg.2024.1498900

**Published:** 2024-10-23

**Authors:** Zoe Sirotiak, Duck-chul Lee, Angelique G. Brellenthin

**Affiliations:** ^1^Department of Kinesiology, Iowa State University, Ames, IA, United States; ^2^Department of Health and Human Development, University of Pittsburgh, Pittsburgh, PA, United States

**Keywords:** long COVID, post-acute sequalae of COVID-19, physical activity, exercise, physical health, mental health

## Abstract

**Introduction:**

Physical activity (PA) is associated with better perceived health among individuals with chronic conditions. However, PA’s relationship with perceived health in people with long COVID is unclear and may be modified by long COVID symptom burden.

**Methods:**

Participants with self-reported long COVID (*N* = 379) responded to an online survey cross-sectionally assessing PA levels, perceived physical and mental health, and intensity of CDC-defined long COVID symptoms on a 0–100 scale. Linear regression analyses assessed the associations between PA and perceived physical and mental health, after accounting for sociodemographic, health behavior, and long COVID intensity variables, with *post-hoc* analyses comparing health across PA levels.

**Results:**

Increasing levels of PA were associated with increases in perceived physical health (*β* = 0.27, *p* < 0.001) and mental health (*β* = 0.19, *p* < 0.001) after accounting for sociodemographic and health behavior variables. PA remained significantly associated with perceived physical health (*β* = 0.15, *p* < 0.001) but not perceived mental health (*β* = 0.09, *p* = 0.067) after the adding long COVID intensity to the model. Insufficiently active and active groups reported significant better physical and mental health than the inactive group (*p*s < 0.05), while the highly active group did not significantly differ from any other group on perceived physical or mental health (*p*s > 0.05). Inactive individuals reported significantly greater long COVID symptom burden compared to each other PA level (*p*s < 0.05).

**Conclusion:**

Higher levels of PA may be associated with better physical health among individuals with long COVID, even after accounting for symptom intensity. However, long COVID symptom intensity may confound the relationship between PA and mental health among individuals with long COVID.

## Introduction

Long COVID, also called post-acute sequalae of COVID-19 (PASC), describes the experience of long-term symptoms following COVID-19 infection (Davis et al., 2023) Up to 20% of those infected with COVID-19 develop long COVID, and estimates suggest that over 65 million individuals are affected worldwide (Davis et al., 2023). Long COVID has been associated with significantly worse physical and mental health compared to the general population without long COVID (Ganesh et al., 2021; Sirotiak et al., 2023, 2024a, 2024b). Symptoms of long COVID are wide-ranging and heterogenous, complicating research and treatment efforts (Aiyegbusi et al., 2021). Long COVID is frequently diagnosed by a process of exclusion, in which other potential causes of symptoms are ruled out prior to diagnosis (Koc et al., 2022; Davis et al., 2023; Srikanth et al., 2023). Treatment options for long COVID are limited given the unclear nature of the condition and heterogenous symptom presentation (Koc et al., 2022; Davis et al., 2023). Somatic syndromes, such as fibromyalgia, myalgic encephalomyelitis/chronic fatigue syndrome (ME/CFS), and irritable bowel syndrome, describe collections of physiological symptoms that cannot be attributed to a clear organic cause (Barsky and Borus, 1999; Salari et al., 2022; Savin et al., 2023; Ursini et al., 2023). Active management strategies such as exercise and psychotherapy are common treatment strategies for these conditions, which have emerged as common comorbidities of long COVID (Henningsen, 2018; Salari et al., 2022; Savin et al., 2023; Ursini et al., 2023). Given the unclear cause of long COVID and lack of medical treatment options available, identifying self-management strategies may be particularly important to those living with long COVID.

Physical activity (PA) is one treatment strategy in the context of somatic syndromes, including long COVID, with unclear results (Twisk and Maes, 2009; Johannesson et al., 2015; Vink and Vink-Niese, 2018; Larun et al., 2019; White and Etherington, 2021). Long COVID groups undergoing exercise intervention have reported improved dyspnea, anxiety, kinesiophobia (fear of PA), muscle strength, walking capacity, quality of life, and perceived fatigue (Fugazzaro et al., 2022; Araújo et al., 2023). However, in a cross-sectional retrospective study investigating the relationship between long COVID symptoms and PA, 75% of the sample noted that PA worsened their symptoms, while just 1% reported improved symptoms following PA (Wright et al., 2022). Particularly among individuals who were physically active prior to COVID-19, a lack of ability to return to prior athletic abilities has been related to depression, suicidal thoughts, and desperation regarding a return to PA (Humphreys et al., 2021; Shelley et al., 2021). While 84% of individuals with long COVID reported meeting the PA guidelines prior to long COVID, only 8% met them at present (Wright et al., 2022).

Most studies concerning PA and long COVID have specifically considered the impact of PA on long COVID symptoms, rather than identifying the associations between PA and perceived health status (Humphreys et al., 2021; Shelley et al., 2021; Wright et al., 2022). While long COVID symptom status may be a useful consideration in tracking condition progression, perceived health may provide better information regarding the impact to the lived experience and functional abilities of each individual affected. Health perception, defined as an individual’s beliefs regarding their health status, describes to what degree an individual feels they can function in physical, mental, and social domains (Kaleta et al., 2009). By considering an individual’s quality of life and functional abilities, rather than just their physiological status or symptoms, the recent consideration of perceived health in medical research has been heralded as a meaningful and impactful change to health-related measurement in a variety of populations (Broderick et al., 2013). Therefore, the purpose of this study is to investigate the association of PA and symptom intensity with perceived physical and mental health among individuals with long COVID.

## Method

A total of 379 adults in the United States (US) with self-reported long COVID responded to an online survey inquiring about sociodemographic characteristics, health status, psychological traits, and experiences during the COVID-19 pandemic. Participants were asked the number of times they had experienced COVID-19, either confirmed by a lab test (PCR, antigen, or other) or assumed by a healthcare provider or themselves. Individuals endorsing at least one infection were then asked whether they experienced long-term symptoms following COVID-19 infection. Participants endorsing currently experiencing long-term symptoms following COVID-19 infection were considered to have long COVID. Participants were not required to report symptoms for a specific duration of time due to the variation in the duration labeled long-term symptoms of COVID-19; instead, long COVID status was self-reported. Inclusion criteria included being over the age of 18 and US-dwelling. Participants were recruited through mass email to students, staff, faculty, and alumni at a large university, as well as posts on long COVID-specific social media sites. The study was approved by the Iowa State University Institutional Review Board and participants indicated consent prior to entering the survey.

### Measures

#### Participant characteristics

Respondents reported age, gender identity, racial and ethnic groups, highest level of education, and annual income.

#### Long COVID symptom intensity

Participants endorsed long COVID symptoms currently experienced from a list of long COVID symptoms offered by the CDC (Center for Disease Control and Prevention, 2022). Participants were presented with a 0–100 scale and asked to rate the intensity of each endorsed symptom, with higher scores associated with greater severity of that symptom. Symptoms were grouped as defined by the CDC (Center for Disease Control and Prevention, 2022) into five categories: (1) general symptoms: exhaustion, tiredness, or fatigue; fever; symptoms that worsen after physical or mental effort; (2) cardiorespiratory symptoms: difficulty breathing or shortness of breath; cough; chest pain or tightness; fast heart rate or heart palpitations; (3) neurological symptoms: difficulty thinking, concentrating, or brain fog; pins-and-needles feeling (nerve pain); headache; lightheadedness or dizziness; sleep problems; changes in smell or taste; depression; anxiety; (4) digestive symptoms: abdominal or stomach pain; diarrhea; and (5) other symptoms: joint or muscle pain; rash; changes in menstrual cycle. Overall intensity ratings represented the combined symptom intensity, with the intensity of individual symptoms reported summed. Each subcategory of symptoms was also assigned an average intensity rating of endorsed symptoms within each symptom category.

#### Perceived physical and mental health

Perceived physical and mental health were measured with the Patient Reported Outcomes Measurement Information System (PROMIS) Global Health, version 1.2 (National Institutes of Health, 2019). Two subscale scores can be calculated from the full scale, resulting in physical and mental health subscales. Each subscale consisted of four items. Physical health questions included a question about general physical health, ability to carry out activities of daily living, average pain, and average fatigue. Mental health questions included a question about quality of life, general mental health including mood and thinking ability, satisfaction with social activities and relationships, and emotional problems. Raw scores for each subscale were converted to *T*-scores, with a mean of 50 and a standard deviation of 10, as indicated by established values for the US adult population (Northwestern University, 2023). Higher scores indicate better perceived physical and mental health. Internal consistency within the current sample was as follows: physical health *α* = 0.59 (*α* = 0.69 among individuals with no missingness); mental health *α* = 0.67 (*α* = 0.75 among individuals with no missingness). This scale has been utilized in similar chronic illness populations (Merriwether et al., 2016; Pak et al., 2021).

#### Physical activity level

Moderate (e.g., brisk walking) or vigorous (e.g., running) aerobic PA level was measured by inquiring about the number of minutes in an average week each participant engages in moderate or vigorous aerobic PA. Participants selected from four provided options: 0 min; 1 to <150 min; 150 to <300 min; and ≥ 300 min per week. Responses fit into established categories based on the 2018 Physical Activity Guidelines for Americans (0 min = inactive; 1 to <150 min = insufficiently active; 150 to <300 min = active; ≥300 min = highly active) (U.S. Department of Health and Human Services, 2018).

#### Alcohol consumption

Alcohol intake was measured, which was then converted into four categories of alcohol consumption: never, former, moderate, and excessive. Moderate alcohol consumption was considered to be 14 drinks or less per week for men, or 7 drinks or less per week for women (U.S. Department of Agriculture and U.S. Department of Health and Human Services, 2020). Excessive alcohol consumption was considered to more than 14 drinks per week for men or more than 7 drinks per week for women.

#### Smoking status

Participants reported whether they currently smoke cigarettes, chew tobacco, vape, or use e-cigarettes. Responses were coded dichotomously: “yes” or “no.”

#### Sleep quality

Sleep quality was assessed with the Patient-Reported Outcome Measurement Information System (PROMIS) Sleep Disturbance Short Form 4a (PROMIS Health Organization, 2021). Respondents rate 4 items, two of which are reverse coded, on a 5-point Likert scale. Scores range from 4 to 20, with higher scores indicating greater sleep disturbance. Raw scores were translated into established *T*-scores for each participant, as recommended (PROMIS Health Organization, 2021). Internal consistency in the current sample was *α* = 0.62 (*α* = 0.69 among individuals with no missingness). The PROMIS sleep measures have been utilized in similar chronic condition populations (Burgess et al., 2019; Chimenti et al., 2021).

### Statistical analyses

Statistical analyses were conducted in SPSS, version 27 (IBM Corp, 2020). There were no imputations performed on any scale. Sociodemographic variables (age, gender identity, racial and ethnic group, education, and income) that have been associated with long COVID were included in the regression analyses as covariates (Perlis et al., 2022; Jacobs et al., 2023). Alcohol use, smoking status, and sleep disturbance were also included as covariates due to their association with long COVID (Wickham et al., 2020; Paul and Fancourt, 2022; Subramanian et al., 2022). Gender identity, racial group, and ethnic group were dichotomized (male/female, white/non-white, not Hispanic or Latino/Hispanic or Latino) to reduce categories with a low number of participants.

Multivariable linear regression models were used to assess the association between PA level and perceived physical and mental health. Model 1 consisted of current PA level, while Model 2 added sociodemographic characteristics (age, gender identity, racial and ethnic group, education, and income). Model 3 added health behaviors (alcohol use, smoking status, sleep disturbance). *Post-hoc* comparison tests with Bonferroni adjustment assessed differences in perceived health among PA levels after accounting for sociodemographic and health behavior variables. Model 4 considered intensity of long COVID symptoms. A linear regression model with Bonferroni-adjusted *post-hoc* comparisons assessed differences in average intensity ratings for CDC-defined groupings of symptoms by PA level. Individuals with missing variables in the model were removed from that analysis. A significant level of *p* < 0.05 was utilized for each analysis.

## Results

Participants were an average 41.9 (SD = 16.6) years old, and most identified as female (53.3%), white (87.9%) and not Hispanic or Latino (85.2%). Most of the sample had received at least a bachelor’s degree (63.8%). More than half participants noted moderate alcohol intake (58.0%) and no smoking (68.3%). The sample reported half a standard deviation greater sleep disturbance than the U.S. general population (*T* = 55.4), and perceived physical and mental health one standard deviation worse than the U.S. general population (*T*s = 39.7, 41.4, respectively) (PROMIS Health Organization, 2021; Northwestern University, 2023). Around half (47.5%) of the participants reported being insufficiently active. Detailed sociodemographic characteristics can be viewed in Table 1.

**Table 1:** Participant characteristics (*N* = 379).

	*N* (%)
Age M years (SD)	41.9 (16.6)
Missing	60 (15.8)
Gender identity, *N* (%)	
Male	167 (44.1)
Female	202 (53.3)
Transgender woman	3 (0.8)
Transgender man	3 (0.8)
Genderqueer/Gender-nonconforming	4 (1.1)
Racial group, *N* (%)	
White	333 (87.9)
Non-white	46 (12.1)
Ethnic group, *N* (%)	
Hispanic or Latino	43 (11.3)
Not Hispanic or Latino	323 (85.2)
Prefer not to answer/Unknown	13 (3.4)
Education, *N* (%)	
Less than high school	8 (2.1)
High school	50 (13.2)
Associate/technical degree	79 (20.8)
Bachelor’s degree	152 (40.1)
Graduate school/professional	90 (23.7)
Income, *N* (%)	
Less than $16,000	41 (10.8)
$16,000 – $34,999	47 (12.4)
$35,000 – $49,999	30 (7.9)
$50,000 – $74,999	70 (18.5)
$75,000 – $99,999	59 (15.6)
Greater than $100,000	123 (32.5)
Unsure	9 (2.4)
Sleep quality, M (SD)	55.4 (6.7)
Missing	4 (1.1)
Alcohol consumption, *N* (%)	
Never	51 (13.5)
Former	95 (25.1)
Moderate	220 (58.0)
Excessive	13 (3.4)
Currently smoking, *N* (%)	
No	259 (68.3)
Yes	120 (31.7)
Perceived Health, M T-score (SD)	
Physical health	39.7 (7.8)
Missing, *N* (%)	1 (0.3)
Mental health	41.4 (8.1)
Missing, *N* (%)	1 (0.3)
Physical Activity, *N* (%)	
Inactive	80 (21.1)
Insufficiently active	180 (47.5)
Active	93 (24.5)
Highly active	26 (6.9)
Combined LC intensity M (SD)	362.3 (317.2)
Not reported/Missing, *N* (%)	64 (16.9)
Average LC intensity per symptom group, M (SD)	
General	64.0 (23.1)
Not reported/Missing, *N* (%)	139 (36.7)
Cardiorespiratory	53.1 (23.5)
Not reported/Missing, *N* (%)	167 (44.1)
Neurological	62.9 (20.5)
Not reported/Missing, *N* (%)	129 (34.0)
Digestive	54.6 (26.0)
Not reported/Missing, *N* (%)	307 (81.0)
Other	63.1 (22.0)
Not reported/Missing, *N* (%)	230 (60.7)

^a^LC, long COVID; M, mean; N, number.

^b^Physical and mental health assessed through the PROMIS Global Health v1.2 scale, with converted T-scores reported.

^c^Sleep was measured by the PROMIS Sleep Disturbance Short Form 4a, with higher scores indicating increased sleep disturbance.

^d^Current alcohol consumption categories as determined by the Dietary Guidelines for Americans, 2020–2025.

^e^Physical activity reported via self-report of moderate or vigorous physical activity (inactive = 0 min; insufficiently active = 1 to < 150 min, active = 150 to < 300 min, highly active = ≥ 300 min).

^f^Not reported/Missing, CDC symptom or symptom group not reported or answer missing.

A linear regression model demonstrated that PA level was significantly positively associated with perceived physical health (Model 1; *β* = 0.35, *t*(281) = 6.20, *p* < 0.001). PA level remained significantly associated with perceived physical health after accounting for sociodemographic variables (Model 2; *β* = 0.32, *t*(281) = 5.80, *p* < 0.001) and health behaviors (Model 3; *β* = 0.27, *t*(281) = 5.34, *p* < 0.001). The total intensity of CDC-defined long COVID symptoms significantly contributed to the model (Model 4; *β* = −0.48, *t*(281) = −9.85, *p* < 0.001). However, the association between PA level and perceived physical health remained significant after accounting for long COVID intensity (Model 4; *β* = 0.15, *t*(281) = 3.44, *p* < 0.001). In the sensitivity analysis, the interaction of PA and long COVID intensity on perceived physical health was not significant (*β* = −0.11, *t*(281) = −1.06, *p* = 0.292). Detailed results can be viewed in Table 2.

**Table 2:** Multivariable linear regression of PA level and long COVID symptom intensity on physical health.

	Model 1			Model 2			Model 3			Model 4		
	*B* (SE)	*β*	*p*-value	*B* (SE)	*β*	*p-*value	*B* (SE)	*β*	*p-*value	*B* (SE)	*β*	*p-*value
PA level	3.53 (0.57)	0.35	**<0.001**	3.30 (0.57)	0.32	**<0.001**	2.70 (0.51)	0.27	**<0.001**	1.55 (0.45)	0.15	**<0.001**
Age (years)	–	–	–	−0.00 (0.03)	−0.00	0.981	−0.06 (0.03)	−0.12	0.052	−0.05 (0.03)	−0.10	0.065
Gender identity	–	–	–	−1.89 (0.92)	−0.12	**0.041**	−1.92 (0.84)	−0.12	**0.023**	−0.20 (0.74)	−0.01	0.786
Racial group	–	–	–	−1.21 (1.34)	−0.05	0.368	−1.26 (1.18)	−0.05	0.286	−1.33 (1.01)	−0.06	0.190
Ethnic group	–	–	–	−0.65 (1.30)	−0.03	0.618	−0.66 (1.14)	−0.03	0.563	−0.71 (0.98)	−0.03	0.469
Education	–	–	–	0.10 (0.45)	0.01	0.820	−0.44 (0.41)	−0.06	0.280	−0.49 (0.35)	−0.07	0.164
Income	–	–	–	0.64 (0.27)	0.14	**0.020**	0.46 (0.24)	0.10	0.061	0.31 (0.21)	0.07	0.144
Alcohol use	–	–	–	–	–	–	0.86 (0.55)	0.08	0.117	−0.01 (0.48)	−0.00	0.985
Smoking status	–	–	–	–	–	–	−3.36 (1.02)	−0.19	**0.001**	−3.56 (0.88)	−0.20	**<0.001**
Sleep disturbance	–	–	–	–	–	–	−0.49 (0.06)	−0.42	**<0.001**	−0.34 (0.05)	−0.29	**<0.001**
LC intensity	–	–	–	–	–	–	–	–	–	−0.01 (0.00)	−0.48	**<0.001**
Overall Adjusted *R*^2^	0.12			0.14			0.34			0.51		
Overall model *p*-value	<0.001			<0.001			<0.001			<0.001		

^a^LC, long COVID; B, unstandardized coefficient; SE, standard error; β, standardized coefficient.

^b^Physical health assessed through the PROMIS Global Health v1.2 scale.

^c^Sleep was measured by the PROMIS Sleep Disturbance Short Form 4a.

^d^Alcohol consumption categorized as determined by the Dietary Guidelines for Americans, 2020–2025.

^e^Physical activity reported via self-report of moderate or vigorous physical activity (inactive = 0 min; insufficiently active = 1 to < 150 min, active = 150 to < 300 min, highly active = ≥ 300 min).

^f^LC intensity refers to the summed intensity of CDC-defined long COVID symptoms endorsed by participants, with endorsed symptoms rated on a 0–100 scale.

^g^Variables coded as detailed in Table 1.

^h^Bolded results significantly contributed to the model.

A linear regression model demonstrated that PA level was significantly associated with perceived mental health (Model 1; *β* = 0.28, *t*(281) = 4.94, *p* < 0.001). PA level continued to remain significantly associated with perceived mental health after accounting for sociodemographic variables (Model 2; *β* = 0.25, *t*(281) = 4.58, *p* < 0.001) and health behaviors (Model 3; *β* = 0.19, *t*(281) = 3.77, *p* < 0.001). The total intensity of CDC-defined long COVID symptoms significantly contributed to the model (Model 4; *β* = −0.45, *t*(281) = −8.74, *p* < 0.001). The association between PA level and perceived mental health was no longer significant after accounting for long COVID intensity (Model 4; *β* = 0.09, *t*(281) = 1.84, *p* = 0.067). In the sensitivity analysis, the interaction of PA and long COVID intensity on perceived mental health was not significant (*β* = −0.10, *t*(281) = −0.90, *p* = 0.369). Detailed regression results can be found in Table 3.

**Table 3:** Multivariable linear regression of PA level and long COVID symptom intensity on mental health.

	Model 1			Model 2			Model 3			Model 4		
	*B* (SE)	*β*	*p*-value	*B* (SE)	*β*	*p-*value	*B* (SE)	*β*	*p-*value	*B* (SE)	*β*	*p-*value
PA level	2.92 (0.59)	0.28	**<0.001**	2.57 (0.56)	0.25	**<0.001**	2.00 (0.53)	0.19	**<0.001**	0.89 (0.49)	0.09	0.067
Age (years)	–	–	–	0.11 (0.03)	0.23	**<0.001**	0.09 (0.03)	0.17	**0.006**	0.10 (0.03)	0.19	**<0.001**
Gender identity	–	–	–	−2.87 (0.91)	−0.17	**0.002**	−2.50 (0.88)	−0.15	**0.005**	−0.86 (0.80)	−0.05	0.287
Racial group	–	–	–	−0.02 (1.33)	−0.00	0.986	0.16 (1.24)	0.01	0.899	0.09 (1.09)	0.00	0.934
Ethnic group	–	–	–	−1.60 (1.28)	−0.07	0.215	−1.39 (1.20)	−0.06	0.246	−1.44 (1.06)	−0.06	0.175
Education	–	–	–	−0.30 (0.45)	−0.04	0.511	−0.62 (0.42)	−0.08	0.144	−0.67 (0.38)	−0.09	0.077
Income	–	–	–	0.64 (0.27)	0.14	**0.018**	0.57 (0.26)	0.13	**0.027**	0.42 (0.23)	0.09	0.063
Alcohol use	–	–	–	–	–	–	1.16 (0.58)	0.10	**0.044**	0.33 (0.52)	0.03	0.526
Smoking status	–	–	–	–	–	–	−0.60 (1.07)	−0.03	0.576	−0.79 (0.95)	−0.05	0.402
Sleep disturbance	–	–	–	–	–	–	−0.39 (0.06)	−0.33	**<0.001**	−0.25 (0.06)	−0.21	**<0.001**
LC intensity	–	–	–	–	–	–	–	–	–	−0.01 (0.00)	−0.45	**<0.001**
Overall Adjusted *R*^2^	0.08			0.18			0.30			0.45		
Overall model *p*-value	<0.001			<0.001			<0.001			<0.001		

^a^LC, long COVID; B, unstandardized coefficient; β, standardized coefficient.

^b^Physical health assessed through the PROMIS Global Health v1.2 scale.

^c^Sleep was measured by the PROMIS Sleep Disturbance Short Form 4a.

^d^Alcohol consumption categorized as determined by the Dietary Guidelines for Americans, 2020–2025.

^e^Physical activity reported via self-report of moderate or vigorous physical activity (inactive = 0 min; insufficiently active = 1 to < 150 min, active = 150 to < 300 min, highly active = ≥ 300 min).

^f^LC intensity refers to the summed intensity of CDC-defined long COVID symptoms endorsed by participants, with endorsed symptoms rated on a 0–100 scale.

^g^Variables coded as detailed in Table 1.

^h^Bolded results significantly contributed to the model.

**Figure 1:**
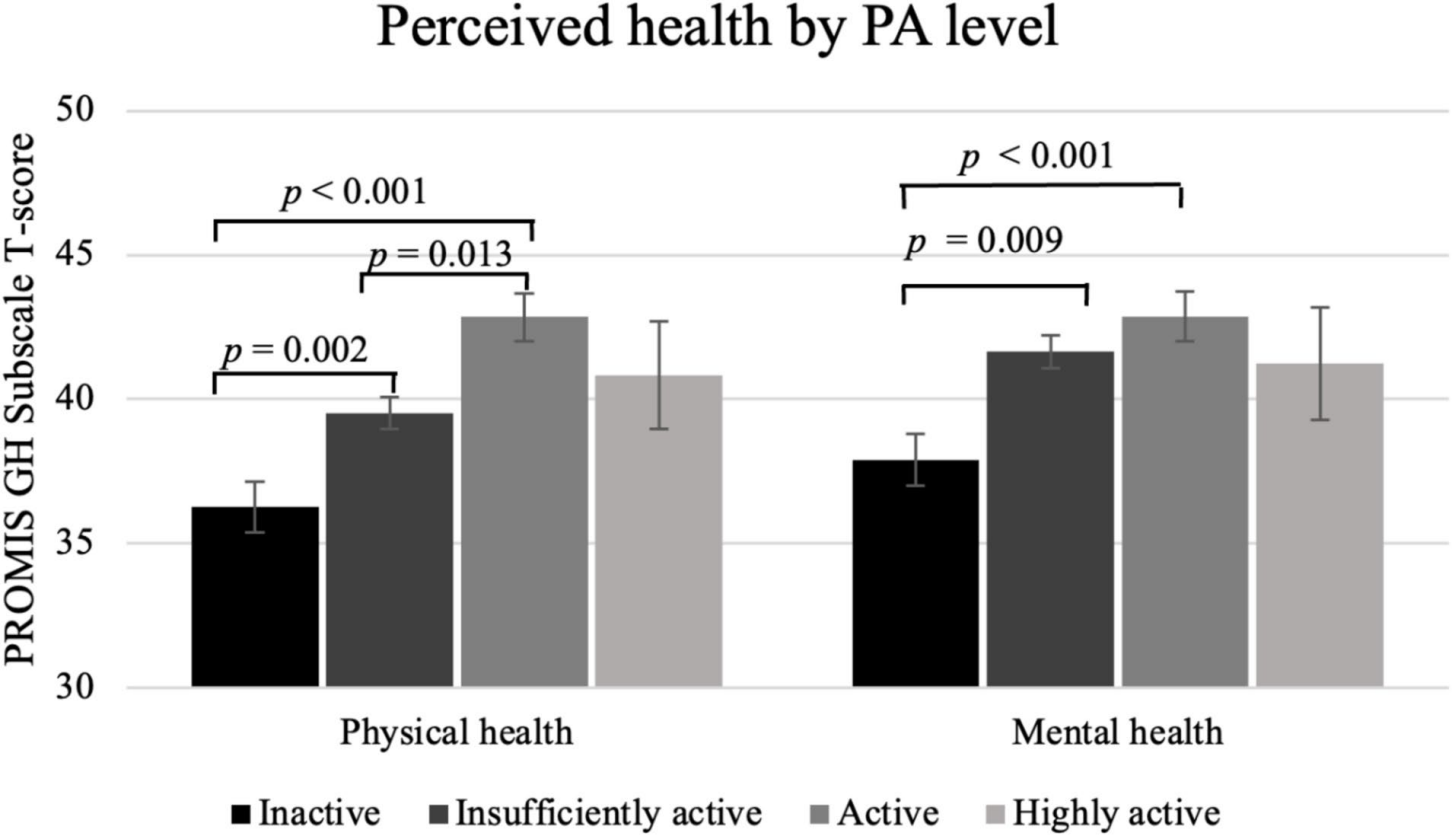
Perceived physical and mental health by PA level among individuals with long COVID. Physical health assessed through the PROMIS Global Health v1.2 scale. Physical activity reported via self-report of moderate or vigorous physical activity (inactive = 0 min; insufficiently active = 1 to <150 min, active = 150 to <300 min, highly active = ≥300 min). Estimated marginal means shown, adjusted for age, gender identity, racial and ethnic group, education, income, alcohol consumption, current smoking status, and sleep disturbance. Standard error bars shown.

*Post-hoc* comparison tests with Bonferroni adjustment (Model 3) were utilized to determine estimated marginal means of perceived physical health *T*-score by PA level, M(SE): inactive: 36.1 (0.9); insufficiently active: 39.7 (0.5); active: 42.8 (0.8); highly active: 41.1 (1.8). The comparison tests with Bonferonni adjustment demonstrated that the inactive group reported significantly worse perceived physical health than the insufficiently active (*p =* 0.002) and active (*p* < 0.001) groups. The insufficiently active group had significantly worse perceived physical health than the active group (*p* = 0.013). The highly active group did not significantly differ from any of the other PA levels (*p*s > 0.05), in part due to small sample size. *Post-hoc* comparison tests with Bonferroni adjustment (Model 3) were utilized to determine estimated marginal means of perceived mental health *T*-score by PA level, M(SE): inactive: 38.1 (0.9); insufficiently active: 41.6 (0.6); active: 43.0 (0.9); highly active: 42.0 (1.9). The comparison tests with Bonferonni adjustment showed the inactive group had significantly worse perceived mental health than the insufficiently active (*p =* 0.009) and active (*p* < 0.001) groups. The highly active group did not significantly differ in their mental health from any other activity group (*p* > 0.05). Figure 1 demonstrates these relationships among PA levels.

**Figure 2:**
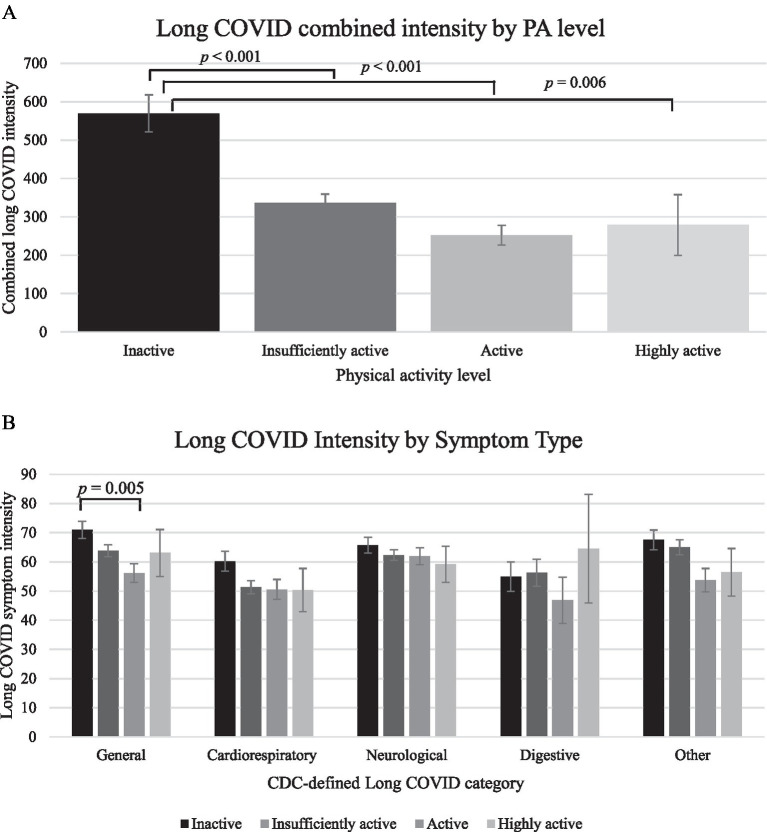
(a) Long COVID combined intensity by PA level. (b) Long COVID intensity in each CDC-defined symptom group by PA level. Mean intensity shown. Physical activity reported via self-report of moderate or vigorous physical activity (inactive = 0 min; insufficiently active = 1 to <150 min, active = 150 to <300 min, highly active = ≥ 300 min). Long COVID symptom groups are as defined by the CDC. All comparisons not noted as significant had *p*-values >0.05 following Bonferroni adjustment. Standard error bars shown.

Combined symptom intensity, considering all CDC-defined long COVID symptoms, was significantly higher among inactive individuals. The inactive group reported significantly greater combined long COVID intensity compared to the insufficiently active (*p* < 0.001), active (*p* < 0.001), and highly active (*p* = 0.006) groups following Bonferroni adjustment. However, no other comparisons among PA levels were significant (*p*s > 0.05). Overall intensity ratings by PA level can be viewed in Figure 2. The average intensity within each CDC-defined symptom category generally showed the highest intensity rating across symptom categories among the inactive group, except for the digestive symptom category, which had the highest intensity rating among the highly active group. However, the only significant comparison among symptom groups by PA level was significantly greater general long COVID intensity among the active group compared to the inactive group (*p* = 0.005). Average intensity symptom ratings by symptom group and PA level can be viewed in Figure 2b.

## Discussion

The significant association between PA level and perceived physical health among individuals with long COVID fits with literature noting improvements in physical health with increasing PA levels among both control and long COVID samples (Anderson and Durstine, 2019; Fugazzaro et al., 2022; Araújo et al., 2023). Similar to the PA and perceived physical health association, PA was significantly associated with perceived mental health among the sample, fitting with PA intervention studies in the context of long COVID (Araújo et al., 2023). Long COVID intensity was significantly associated with both perceived physical and mental health after accounting for sociodemographic and health behaviors, including PA level. PA level continued to be significantly associated with perceived physical health after accounting for long COVID intensity, further supporting the idea that PA appears to be uniquely associated with physical health in long COVID beyond sociodemographic, illness severity, and health behavior factors (Humphreys et al., 2021; Wright et al., 2022; Colas et al., 2023).

Increasing PA levels were generally associated with increasing levels of perceived health, fitting with literature noting better physical and mental health ratings among individuals with long COVID engaging in more PA (Vélez-Santamaría et al., 2023). Comparisons involving the inactive category particularly stood out, with insufficiently active and active groups reporting significantly greater perceived physical and mental health scores than inactive individuals. These results suggest that any amount of PA within the comfort zone and ability level of individuals with long COVID may provide physical and mental health benefits, although prospective data are required. The consistent increases in perceived physical and mental health with increasing PA level fits with established literature in both healthy and clinical samples demonstrating that even a small volume of PA can have measurable health benefits (Blair et al., 1992; Huber et al., 2020; Centers for Disease Control and Prevention, 2023b).

Although the insufficiently active and active groups reported better perceived physical and mental health than inactive individuals, comparisons of other PA levels with the highly active group were not significant in both perceived physical and mental health analyses, conflicting with findings in the general population showing that each increased activity level is associated with better perceived health (Tyson et al., 2010). It is unclear why there was a tapering of perceived health observed among the highly active group, but there are several possible explanations. Post-exertional malaise, or worsened symptoms following physical, mental, or emotional exertion, can affect both physical and mental health and it has been noted following PA among individuals with long COVID (Stussman et al., 2020; Humphreys et al., 2021; Vernon et al., 2023; Appelman et al., 2024). It is possible that high levels of PA, while providing some perceivable health benefits, may also be associated with post-exertional malaise, attenuating perceived health. However, post-exertional malaise is often noted following even low intensity and duration of exertion among individuals affected (Centers for Disease Control and Prevention, 2023a), and it is unclear whether or why individuals reporting high levels of activity may be particularly prone in this sample. Symptomatic responses to PA were also not assessed in our sample, complicating the consideration of response to exercise and post-exertional malaise among PA groups.

Regarding the lack of significant perceived mental health comparisons among the highly active group, individuals who were physically active before COVID-19, such as living an active lifestyle or being a competitive athlete, have reported significant losses of identity associated with long COVID development and loss of abilities (Humphreys et al., 2021; Shelley et al., 2021; Wurz et al., 2022). It is possible that individuals who were physically active before COVID-19 may continue to attempt high levels of PA, becoming frustrated when their physical ability limitations become apparent (Humphreys et al., 2021; Shelley et al., 2021; Wurz et al., 2022), leading to the lack of improved perceived mental health noted among highly active individuals with long COVID. Observational studies among long COVID samples have reported conflicting associations between PA and mental health, with some participants reporting improved mental health and cognitive functioning with PA, while others report that their symptoms worsen (Humphreys et al., 2021; Shelley et al., 2021; Wright et al., 2022). Illness perception, coping mechanisms, and previous mental health status may each affect how an individual understands and manages their condition, affecting their mental health, thus offering other explanations for the results that were not accounted for in the regression analyses (Turner and Kelly, 2000; Petrie et al., 2008; Algorani and Gupta, 2023). The relatively low number of individuals with long COVID reporting being highly active (*N* = 26; 6.9%) compared to the other PA level groups is another important consideration. The highly active group reported greater variation in perceived health compared to the other PA levels, with over double the standard error of the second most variable PA level in both the physical health (SE = 1.83), and mental health (SE = 1.96) models. Therefore, it appears that individuals reporting both relatively low and high ratings of perceived health (physical health range: *T* = 26.7–54.1; mental health range: *T* = 28.4–62.5) may have been included in this PA level group, creating a wide distribution of perceived health scores within the highly active group and contributing to the lack of significant comparisons observed.

Long COVID severity is another important consideration in understanding perceived health. Individuals with severe ME/CFS are often bedbound for extensive periods of time, limiting most PA including activities of daily living (Chang et al., 2021; Montoya et al., 2021). Long COVID appears to follow a similar course, with severely affected individuals limited in most aspects of daily life (Brodin et al., 2022; Davis et al., 2023). Severely ill individuals with ME/CFS have noted significant health burden (Chang et al., 2021; Ziauddeen et al., 2022), including fatigue, pain interference, and pain behavior. Given the strong associations between ME/CFS and long COVID (Komaroff and Lipkin, 2023), it seems plausible that long COVID severity may be driving the association noted between PA levels and perceived physical and mental health (i.e., people with severe symptoms may be more likely to be inactive and have worse perceived health). Following the addition of combined long COVID severity to the model, however, current PA level remained significantly associated with perceived physical health (*p* < 0.001), but not perceived mental health (*p* = 0.067). The few significant differences among PA groups in average severity by CDC-defined symptom category further suggest that PA appears to be uniquely associated with perceived physical health, suggesting that PA promotion, even to levels below the current PA guidelines, may be helpful in enhancing physical health in the context of long COVID.

Although PA level continued to be associated with perceived physical health after accounting for long COVID intensity, long COVID intensity was a stronger predictor of perceived health. Long COVID intensity had an absolute standardized coefficient more than three times greater than that of PA in predicting perceived physical health (*β* = −0.48 and 0.15, respectively), and an absolute standardized coefficient more than four times greater than that of PA in predicting mental health (*β* = −0.45 and 0.09, respectively). This result seems to fit with literature noting that perceived severity of illness is negatively associated with perceived health in clinical contexts (Lackner et al., 2014; Kim et al., 2015). Therefore, while PA level appears to be positively associated with perceived physical and mental health, the intensity of long COVID symptoms experienced appears to be a more impactful predictor. Increasing PA levels, particularly from inactive to at least some PA may be a useful long COVID management technique, however, our results indicate that targeting long COVID intensity, and ensuring that PA does not worsen symptoms, must be considered as well. Strategies previously suggested in the context of other somatic syndromes include treating the most disruptive symptoms first and identifying treatment strategies that target multiple symptoms for maximum benefit (Centers for Disease Control and Prevention, 2021).

This study had limitations. First, the survey offered a cross-sectional assessment of long COVID and PA, as well as perceived physical and mental health, and causation and direction of association cannot be inferred. The survey relied on participant self-report for diagnosis of COVID-19, though it has been reported that some who report prior COVID-19 infection do not have COVID-19 antibodies (Matta et al., 2022). PA status was also self-reported, and muscle strengthening PA was not assessed. Moderate and vigorous intensity PA were not individually assessed, and the PA level assignment of individuals may vary from the 2018 Physical Activity Guidelines for Americans, which doubles vigorous minutes in the determination of moderate-to-vigorous PA level (U.S. Department of Health and Human Services, 2018). In addition, limited medical history was obtained, and it is unclear how unmeasured physical comorbidities might have affected the results. The inclusion and exclusion criteria, as well as the recruitment strategies utilized in the study may have affected those who participated in the study, limiting generalizations and conclusions that can be drawn from the data. Although this study consisted of largely of individuals with white and not Hispanic or Latino identities, as well as those with high educational attainment and higher annual income, individuals with not white and Hispanic or Latino identities, as well as individuals with lower educational attainment and lower annual income have been suggested to be more likely to develop long COVID and be more severely affected (Wong and Weitzer, 2021; Jacobs et al., 2023; Tanne, 2023). No official definition of long COVID was utilized, with all participants endorsing current long-term symptoms following COVID-19 being considered to have long COVID. However, the prevalence of long COVID in our overall sample was 8.8%, which is smaller than the estimated 10–20% for individuals with COVID-19 infection developing long COVID in the general population (The Lancet, 2023). Therefore, our sample is likely more conservative in identifying those with long COVID. Not all CDC-defined long COVID symptom categories were noted by each individual reporting long COVID, leaving smaller sample sizes in several symptom categories. Finally, while participants were asked about long COVID intensity, frequency and duration of long COVID symptoms were not probed though these factors may also affect perceived long COVID severity.

## Conclusion

PA level was significantly positively associated with both perceived physical and mental health among individuals with long COVID. PA remained associated with perceived physical health even after accounting for the intensity of long COVID symptoms. Individuals reporting insufficiently active or active PA levels each reported significantly better perceived physical and mental health than individuals who reporting inactive PA levels, suggesting that even modest amounts of PA may be associated with significantly better perceived health among individuals with long COVID. However, long COVID symptom intensity appears to be a stronger predictor of perceived physical and mental health than PA, emphasizing the importance of targeting disruptive symptoms in addressing long COVID. This study was cross-sectional, and future PA intervention studies and dose–response studies may be helpful in further elucidating the relationship between PA level and perceived health among individuals with long COVID. In addition, future studies could assess the impact of changing PA level on specific physical and mental health outcomes, such as functional ability level, pain, or clinical depression and anxiety.

## Data Availability

The raw data supporting the conclusions of this article will be made available by the authors, without undue reservation.
